# 1,3-Bis(3-phenyl­prop­yl)benzimidazolium bromide monohydrate

**DOI:** 10.1107/S1600536808030432

**Published:** 2008-09-27

**Authors:** Mehmet Akkurt, Selvi Karaca, Ülkü Yılmaz, Hasan Küçükbay, Orhan Büyükgüngör

**Affiliations:** a Department of Physics, Faculty of Arts and Sciences, Erciyes University, 38039 Kayseri, Turkey; b Department of Chemistry, Faculty of Arts and Sciences, Ínönü University, 44280 Malatya, Turkey; c Department of Physics, Faculty of Arts and Sciences, Ondokuz Mayıs University, 55139 Samsun, Turkey

## Abstract

In the title compound, C_25_H_27_N_2_^+^·Br^−^·H_2_O, the benzimidazole unit is essentially planar, with a maximum deviation of 0.020 (6) Å. The benzimidazole unit makes dihedral angles of 83.6 (3) and 81.0 (3)° with the two terminal phenyl rings. The dihedral angle between the phenyl rings is 58.5 (4)°. In the crystal structure, there are C—H⋯O hydrogen bonds, a C—H⋯π inter­action between a phenyl H atom and the phenyl ring of a neighbouring mol­ecule, and a π–π inter­action [3.512 (3) Å] between the centroids of the five-membered ring and the benzene ring of the benzimidazole unit of an adjacent mol­ecule.

## Related literature

For general background, see: Sakai *et al.* (1989 ▶); Küçükbay *et al.* (2001 ▶, 2003 ▶, 2004 ▶). For a similar structure, see: Akkurt *et al.* (2005 ▶). For related structures, see: Akkurt *et al.* (2004 ▶, 2007 ▶); Karaca *et al.* (2005 ▶); Pınar *et al.* (2006 ▶); Yıldırım *et al.* (2005 ▶).
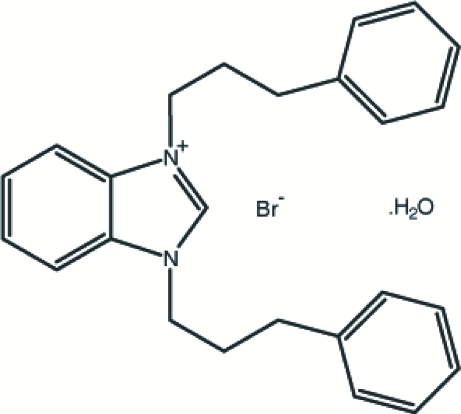


## Experimental

### 

#### Crystal data

C_25_H_27_N_2_^+^·Br^−^·H_2_O
*M* *_r_* = 453.40
Monoclinic, 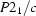

*a* = 14.1933 (8) Å
*b* = 11.4594 (3) Å
*c* = 18.3014 (10) Å
β = 128.916 (3)°
*V* = 2316.1 (2) Å^3^
*Z* = 4
Mo *K*α radiation
μ = 1.79 mm^−1^
*T* = 295 (2) K
0.71 × 0.63 × 0.54 mm

#### Data collection

Stoe IPDS II diffractometer
Absorption correction: integration (**X-RED32**; Stoe & Cie, 2002 ▶) *T*_min_ = 0.363, *T*_max_ = 0.444
18735 measured reflections
5283 independent reflections
2688 reflections with *I* > 2σ(*I*)
*R*_int_ = 0.071

#### Refinement

*R*[*F* ^2^ > 2σ(*F* ^2^)] = 0.065
*wR*(*F* ^2^) = 0.172
*S* = 0.99
5283 reflections
268 parameters
3 restraints
H atoms treated by a mixture of independent and constrained refinement
Δρ_max_ = 0.47 e Å^−3^
Δρ_min_ = −0.23 e Å^−3^

### 

Data collection: *X-AREA* (Stoe & Cie, 2002 ▶); cell refinement: *X-AREA*; data reduction: *X-RED32* (Stoe & Cie, 2002 ▶); program(s) used to solve structure: *SIR97* (Altomare *et al.*, 1999 ▶); program(s) used to refine structure: *SHELXL97* (Sheldrick, 2008 ▶); molecular graphics: *ORTEP-3 for Windows* (Farrugia, 1997 ▶); software used to prepare material for publication: *WinGX* (Farrugia, 1999 ▶).

## Supplementary Material

Crystal structure: contains datablocks global, I. DOI: 10.1107/S1600536808030432/is2337sup1.cif

Structure factors: contains datablocks I. DOI: 10.1107/S1600536808030432/is2337Isup2.hkl

Additional supplementary materials:  crystallographic information; 3D view; checkCIF report

## Figures and Tables

**Table 1:** Hydrogen-bond geometry (Å, °)

*D*—H⋯*A*	*D*—H	H⋯*A*	*D*⋯*A*	*D*—H⋯*A*
C7—H7⋯O1	0.93	2.50	3.257 (10)	139
C17—H17*A*⋯O1	0.97	2.38	3.236 (13)	148
C24—H24⋯*Cg*1^i^	0.93	2.84	3.771 (14)	176

Symmetry code: (i) 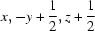. *Cg*1 is the centriod of the C11–C16 phenyl ring.

**Fig. 1.**
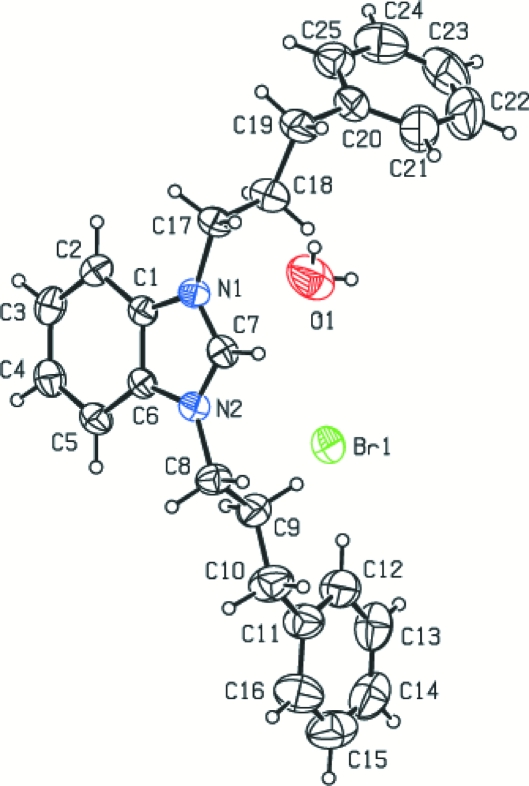
The title molecule (I), with the atom numbering scheme. Displacement ellipsoids for non-H atoms are drawn at the 30% probability level.

**Fig. 2.**
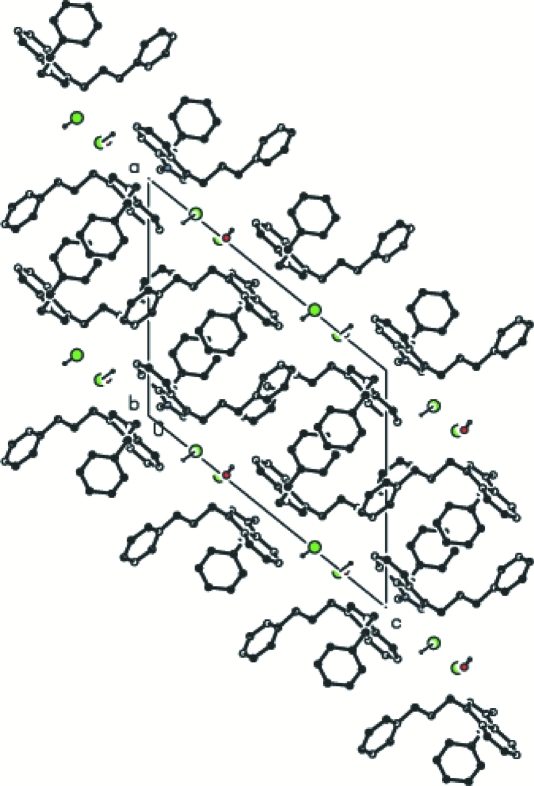
The crystal packing diagram of (I), down the b axis. Dashed lines indicate hydrogen contacts. H atoms not involved in hydrogen bonding have been omitted for clarity.

## supplementary crystallographic information

### Comment

Benzimidazole and related heterocyclic compounds have been extensively investigated because of their versatile pharmacological activity. They are also present in various naturally occurring drugs such as omeprazole, astemizole andemedastine difumarate (Sakai *et al.*, 1989). Substituted benzimidazole moieties are estabilished pharmacophores in parasitic chemotheraphy. In some previous papers (Küçükbay *et al.*, 2001, 2003, 2004), we reported the synthesis and antimicrobial activity of some benzimidazole derivatives. The objective of this study was to elucidate the crystal structure of the title compound, (I).

In the title molecule (I) (Fig. 1), the values of the geometric parameters are comparable with those of previously reported structures (Akkurt *et al.*, 2004, 2005, 2007; Pınar *et al.*, 2006; Yıldırım *et al.*, 2005; Karaca *et al.*, 2005). The benzimidazole unit (N1/N2/C1–C7) is essentially planar, with a maximum deviation of 0.020 (6) Å for C7 from the least-squares plane defined by the nine constituent atoms. The benzimidazole unit makes the dihedral angles of 83.6 (3) and 81.0 (3)° with the two terminal phenyl rings (C11–C16) and (C20–C25), respectively. The dihedral angle between the phenyl rings is 58.5 (4)°.

Molecular conformation is stabilized by intramolecular C—H···O hydrogen bonding interactions. The molecular packing (Fig. 2) is stabilized by a C—H···π interaction between a phenyl H atom and the phenyl ring of neighbouring molecules, with a C24—H24···*Cg*1^ii^ separation of 2.84 Å [Table 1; *Cg*1 is the C11–C16 phenyl ring, symmetry code: (ii) *x*, -*y* + 1/2, *z* + 1/2]. In the crystal packing, there is also a π–π interaction with a distance of 3.512 (3) Å between the centroids of the five-membered ring (N1/N2/C1/C6/C7) (centroid *Cg*2) and the benzene ring (C1–C6) (centroid *Cg*3) of the benzimidazole unit of the adjacent molecule.

### Experimental

A solution of 1-(3-phenylpropyl)benzimidazole (4.20 g,17.80 mmol) and Ph(CH_2_)_3_Br (2.70 ml, 17.85 mmol) was refluxed in DMF for 4 h. The mixture was then cooled and the volatiles were removed from the filtrate *in vacuo*. The residue obtained was then crytallized from EtOH/Et_2_O(1:5) (yield 6.50 g, 84%; m.p. 376–377 K). ^1^H-NMR (DMSO-d_6_): δ (p.p.m.) 2.26 (*p*, CH_2_, 4H), 2.73 (*t*, CH_2_—Ar, 4H), 4.55 (t, CH_2_—N, 4H), 7.14–8.13 (*m*, Ar—H, 14H), 9.99 (s, >CH, 1H). ^13^C-NMR (DMSO-d_6_): δ 30.5, 32.4, 47.0, 114.2, 126.5, 126.9, 128.7, 131.6, 141.4, 142.7. Analysis calculated for C_25_H_29_N_2_OBr: C 66.23, H 6.40, N 6.18%; found: C 65.99, H 6.13, N 6.10%.

### Refinement

Water H atoms were found in a difference Fourier map and distance restraints [O—H = 0.84 (9) and H···H = 1.37 (9) Å] were used to obtain reasonable values for O–H distances and H—O—H angles, with *U*_iso_(H) = 1.5*U*_eq_(O). Other H atoms were positioned to the ideal geometric positions and refined with a riding model, with C—H = 0.93 and 0.97 Å, and with *U*_iso_ = 1.2*U*_eq_(C).

### Crystal data

**Table:**

C_25_H_27_N_2_^+^·Br^−^·H_2_O	*F*(000) = 944
*M**_r_* = 453.40	*D*_x_ = 1.300 Mg m^−^^3^
Monoclinic, *P*2_1_/*c*	Mo *K*α radiation, λ = 0.71073 Å
Hall symbol: -P 2ybc	Cell parameters from 13886 reflections
*a* = 14.1933 (8) Å	θ = 1.4–28.0°
*b* = 11.4594 (3) Å	µ = 1.79 mm^−^^1^
*c* = 18.3014 (10) Å	*T* = 295 K
β = 128.916 (3)°	Block, colourless
*V* = 2316.1 (2) Å^3^	0.71 × 0.63 × 0.54 mm
*Z* = 4	

### Data collection

**Table:**

Stoe IPDS II diffractometer	5283 independent reflections
Radiation source: sealed X-ray tube, 12 x 0.4 mm long-fine focus	2688 reflections with *I* > 2σ(*I*)
plane graphite	*R*_int_ = 0.071
Detector resolution: 6.67 pixels mm^-1^	θ_max_ = 27.6°, θ_min_ = 1.8°
ω scans	*h* = −18→18
Absorption correction: integration (*X-RED32*; Stoe & Cie, 2002)	*k* = −13→14
*T*_min_ = 0.363, *T*_max_ = 0.444	*l* = −23→23
18735 measured reflections	

### Refinement

**Table:**

Refinement on *F*^2^	Primary atom site location: structure-invariant direct methods
Least-squares matrix: full	Secondary atom site location: difference Fourier map
*R*[*F*^2^ > 2σ(*F*^2^)] = 0.065	Hydrogen site location: inferred from neighbouring sites
*wR*(*F*^2^) = 0.173	H atoms treated by a mixture of independent and constrained refinement
*S* = 0.99	*w* = 1/[σ^2^(*F*_o_^2^) + (0.0827*P*)^2^] where *P* = (*F*_o_^2^ + 2*F*_c_^2^)/3
5283 reflections	(Δ/σ)_max_ < 0.001
268 parameters	Δρ_max_ = 0.47 e Å^−^^3^
3 restraints	Δρ_min_ = −0.24 e Å^−^^3^

### Special details

**Table:**

Geometry. Bond distances, angles *etc*. have been calculated using the rounded fractional coordinates. All su's are estimated from the variances of the (full) variance-covariance matrix. The cell e.s.d.'s are taken into account in the estimation of distances, angles and torsion angles
Refinement. Refinement on *F*^2^ for ALL reflections except those flagged by the user for potential systematic errors. Weighted *R*-factors *wR* and all goodnesses of fit *S* are based on *F*^2^, conventional *R*-factors *R* are based on *F*, with *F* set to zero for negative *F*^2^. The observed criterion of *F*^2^ > σ(*F*^2^) is used only for calculating -*R*-factor-obs *etc*. and is not relevant to the choice of reflections for refinement. *R*-factors based on *F*^2^ are statistically about twice as large as those based on *F*, and *R*-factors based on ALL data will be even larger.

### Fractional atomic coordinates and isotropic or equivalent isotropic displacement parameters (Å^2^)

**Table:**

	*x*	*y*	*z*	*U*_iso_*/*U*_eq_	
N1	−0.1093 (3)	0.1691 (3)	0.9193 (2)	0.0541 (10)	
N2	−0.1505 (3)	0.0103 (3)	0.8391 (2)	0.0539 (11)	
C1	−0.1415 (3)	0.0860 (3)	0.9554 (3)	0.0514 (11)	
C2	−0.1469 (4)	0.0912 (4)	1.0285 (3)	0.0622 (14)	
C3	−0.1784 (4)	−0.0099 (4)	1.0477 (3)	0.0753 (17)	
C4	−0.2034 (4)	−0.1121 (4)	0.9973 (3)	0.0742 (17)	
C5	−0.1995 (4)	−0.1173 (3)	0.9243 (3)	0.0645 (16)	
C6	−0.1673 (3)	−0.0157 (3)	0.9049 (3)	0.0505 (11)	
C7	−0.1159 (4)	0.1196 (3)	0.8509 (3)	0.0587 (12)	
C8	−0.1677 (4)	−0.0701 (4)	0.7691 (3)	0.0700 (17)	
C9	−0.3031 (5)	−0.0933 (4)	0.6894 (3)	0.0853 (19)	
C10	−0.3233 (6)	−0.1767 (6)	0.6209 (4)	0.105 (2)	
C11	−0.4522 (5)	−0.2161 (5)	0.5455 (4)	0.0905 (19)	
C12	−0.5522 (5)	−0.1513 (5)	0.5160 (4)	0.102 (2)	
C13	−0.6656 (6)	−0.1874 (7)	0.4419 (4)	0.107 (3)	
C14	−0.6809 (8)	−0.2866 (8)	0.3982 (5)	0.121 (3)	
C15	−0.5849 (10)	−0.3547 (7)	0.4275 (6)	0.128 (4)	
C16	−0.4716 (7)	−0.3209 (6)	0.5006 (6)	0.113 (3)	
C17	−0.0775 (4)	0.2925 (3)	0.9491 (3)	0.0652 (16)	
C18	−0.1947 (5)	0.3644 (4)	0.8951 (4)	0.0844 (19)	
C19	−0.1744 (5)	0.4916 (4)	0.9136 (4)	0.085 (2)	
C20	−0.2942 (5)	0.5556 (4)	0.8583 (4)	0.079 (2)	
C21	−0.3563 (8)	0.5820 (7)	0.7649 (5)	0.126 (3)	
C22	−0.4712 (9)	0.6297 (8)	0.7129 (6)	0.166 (4)	
C23	−0.5191 (7)	0.6575 (6)	0.7529 (8)	0.134 (4)	
C24	−0.4537 (8)	0.6398 (7)	0.8464 (8)	0.132 (4)	
C25	−0.3441 (6)	0.5865 (5)	0.8976 (5)	0.093 (2)	
O1	0.0138 (8)	0.3343 (4)	0.8276 (5)	0.145 (3)	
Br1	0.01170 (5)	0.12751 (4)	0.69968 (4)	0.0869 (2)	
H2	−0.13010	0.15940	1.06230	0.0750*	
H3	−0.18330	−0.01050	1.09600	0.0900*	
H4	−0.22340	−0.17910	1.01360	0.0890*	
H5	−0.21740	−0.18530	0.89000	0.0780*	
H7	−0.09830	0.15740	0.81580	0.0700*	
H8A	−0.12700	−0.14330	0.79930	0.0850*	
H8B	−0.13160	−0.03690	0.74310	0.0850*	
H9A	−0.33960	−0.12290	0.71610	0.1020*	
H9B	−0.34280	−0.02040	0.65790	0.1020*	
H10A	−0.27450	−0.24520	0.65450	0.1260*	
H10B	−0.29360	−0.14250	0.59040	0.1260*	
H12	−0.54230	−0.08240	0.54700	0.1230*	
H13	−0.73240	−0.14220	0.42180	0.1270*	
H14	−0.75850	−0.30960	0.34680	0.1460*	
H15	−0.59700	−0.42500	0.39730	0.1540*	
H16	−0.40610	−0.36840	0.52080	0.1360*	
H17A	−0.02540	0.32160	0.93590	0.0780*	
H17B	−0.03440	0.29890	1.01610	0.0780*	
H18A	−0.24030	0.35110	0.82840	0.1010*	
H18B	−0.24350	0.33690	0.91190	0.1010*	
H19A	−0.12700	0.52050	0.89590	0.1020*	
H19B	−0.12920	0.50610	0.98010	0.1020*	
H21	−0.32100	0.56780	0.73680	0.1510*	
H22	−0.51500	0.64220	0.64880	0.2000*	
H23	−0.59660	0.68890	0.71770	0.1610*	
H24	−0.48460	0.66450	0.87600	0.1580*	
H25	−0.30310	0.57120	0.96090	0.1110*	
HW1	−0.042 (7)	0.364 (8)	0.775 (4)	0.2170*	
HW2	0.071 (6)	0.383 (7)	0.854 (7)	0.2170*	

### Atomic displacement parameters (Å^2^)

**Table:**

	*U*^11^	*U*^22^	*U*^33^	*U*^12^	*U*^13^	*U*^23^
N1	0.0533 (19)	0.0500 (15)	0.0617 (19)	−0.0075 (14)	0.0374 (17)	−0.0040 (15)
N2	0.0536 (19)	0.0543 (17)	0.0612 (19)	−0.0002 (14)	0.0396 (17)	−0.0013 (14)
C1	0.038 (2)	0.0523 (19)	0.054 (2)	0.0024 (16)	0.0242 (18)	0.0012 (17)
C2	0.059 (3)	0.070 (2)	0.058 (2)	−0.002 (2)	0.037 (2)	−0.0056 (19)
C3	0.079 (3)	0.094 (3)	0.065 (3)	0.001 (3)	0.051 (3)	0.010 (2)
C4	0.079 (3)	0.070 (3)	0.080 (3)	−0.003 (2)	0.053 (3)	0.015 (2)
C5	0.067 (3)	0.052 (2)	0.074 (3)	−0.004 (2)	0.044 (2)	0.001 (2)
C6	0.043 (2)	0.0499 (19)	0.057 (2)	0.0042 (16)	0.0307 (19)	0.0045 (17)
C7	0.054 (2)	0.056 (2)	0.069 (2)	−0.0047 (19)	0.040 (2)	0.004 (2)
C8	0.078 (3)	0.071 (3)	0.074 (3)	−0.004 (2)	0.054 (3)	−0.010 (2)
C9	0.087 (4)	0.080 (3)	0.066 (3)	0.013 (3)	0.037 (3)	−0.004 (2)
C10	0.100 (4)	0.116 (4)	0.101 (4)	0.011 (4)	0.064 (4)	−0.021 (4)
C11	0.071 (3)	0.088 (3)	0.089 (4)	−0.002 (3)	0.039 (3)	−0.016 (3)
C12	0.078 (4)	0.099 (4)	0.089 (4)	0.008 (3)	0.033 (3)	−0.010 (3)
C13	0.075 (4)	0.133 (5)	0.091 (4)	−0.012 (4)	0.042 (4)	0.015 (4)
C14	0.116 (6)	0.141 (6)	0.084 (4)	−0.059 (5)	0.052 (4)	−0.009 (4)
C15	0.158 (8)	0.116 (5)	0.138 (6)	−0.047 (6)	0.106 (7)	−0.041 (5)
C16	0.108 (5)	0.099 (4)	0.152 (6)	−0.006 (4)	0.091 (5)	−0.020 (4)
C17	0.068 (3)	0.050 (2)	0.078 (3)	−0.0074 (19)	0.046 (3)	−0.0058 (19)
C18	0.082 (3)	0.067 (3)	0.101 (4)	−0.002 (2)	0.056 (3)	−0.009 (3)
C19	0.093 (4)	0.059 (3)	0.100 (4)	−0.008 (2)	0.059 (3)	−0.006 (2)
C20	0.087 (4)	0.064 (3)	0.090 (4)	0.001 (2)	0.058 (3)	0.006 (2)
C21	0.143 (6)	0.133 (5)	0.111 (5)	0.037 (5)	0.084 (5)	0.026 (4)
C22	0.161 (8)	0.177 (9)	0.109 (6)	0.082 (7)	0.060 (6)	0.045 (5)
C23	0.084 (5)	0.103 (5)	0.174 (8)	0.030 (4)	0.061 (6)	0.019 (5)
C24	0.129 (6)	0.121 (6)	0.174 (8)	0.014 (5)	0.109 (6)	−0.024 (6)
C25	0.104 (4)	0.086 (3)	0.107 (4)	0.002 (3)	0.075 (4)	−0.005 (3)
O1	0.248 (6)	0.096 (3)	0.196 (5)	0.007 (4)	0.190 (5)	0.018 (3)
Br1	0.1042 (4)	0.0715 (3)	0.0851 (4)	−0.0021 (3)	0.0595 (3)	0.0094 (2)

### Geometric parameters (Å, °)

**Table:**

O1—HW1	0.84 (7)	C23—C24	1.355 (16)
O1—HW2	0.84 (11)	C24—C25	1.356 (15)
N1—C1	1.390 (6)	C2—H2	0.9300
N1—C7	1.323 (6)	C3—H3	0.9300
N1—C17	1.480 (5)	C4—H4	0.9300
N2—C6	1.399 (6)	C5—H5	0.9300
N2—C8	1.469 (6)	C7—H7	0.9300
N2—C7	1.313 (5)	C8—H8B	0.9700
C1—C6	1.386 (5)	C8—H8A	0.9700
C1—C2	1.389 (7)	C9—H9B	0.9700
C2—C3	1.365 (7)	C9—H9A	0.9700
C3—C4	1.392 (6)	C10—H10A	0.9700
C4—C5	1.373 (8)	C10—H10B	0.9700
C5—C6	1.376 (6)	C12—H12	0.9300
C8—C9	1.539 (8)	C13—H13	0.9300
C9—C10	1.455 (8)	C14—H14	0.9300
C10—C11	1.511 (10)	C15—H15	0.9300
C11—C12	1.376 (11)	C16—H16	0.9300
C11—C16	1.382 (9)	C17—H17A	0.9700
C12—C13	1.363 (10)	C17—H17B	0.9700
C13—C14	1.327 (12)	C18—H18B	0.9700
C14—C15	1.355 (17)	C18—H18A	0.9700
C15—C16	1.350 (15)	C19—H19B	0.9700
C17—C18	1.535 (9)	C19—H19A	0.9700
C18—C19	1.483 (7)	C21—H21	0.9300
C19—C20	1.514 (10)	C22—H22	0.9300
C20—C25	1.336 (12)	C23—H23	0.9300
C20—C21	1.377 (9)	C24—H24	0.9300
C21—C22	1.384 (16)	C25—H25	0.9300
C22—C23	1.313 (19)		
			
Br1···O1	3.318 (8)	HW2···Br1^iii^	2.96 (8)
Br1···O1^i^	3.383 (5)	HW2···H8B^iii^	2.5800
Br1···HW1^i^	3.04 (9)	H3···Br1^iv^	3.2000
Br1···H17B^ii^	3.1100	H5···C8	3.0200
Br1···H8A^iii^	3.0900	H5···C20^vi^	3.0900
Br1···H3^iv^	3.2000	H5···H9A	2.5900
Br1···HW2^i^	2.96 (8)	H7···O1	2.5000
Br1···H8B	3.2200	H7···H17A	2.5700
Br1···H2^ii^	3.1600	H7···H8B	2.4800
Br1···H13^v^	3.1000	H8A···H10A	2.4100
O1···C17	3.236 (13)	H8A···Br1^i^	3.0900
O1···Br1^iii^	3.383 (5)	H8A···C5	3.0600
O1···C7	3.257 (10)	H8B···H10B	2.5500
O1···Br1	3.318 (8)	H8B···HW2^i^	2.5800
O1···H17A	2.3800	H8B···H7	2.4800
O1···H7	2.5000	H8B···Br1	3.2200
N1···N2	2.175 (5)	H9A···C5	2.9900
N2···N1	2.175 (5)	H9A···C12	2.9600
C1···C6^iv^	3.509 (7)	H9A···H5	2.5900
C2···C8^iv^	3.597 (7)	H9A···C6	2.9700
C3···C7^iv^	3.568 (9)	H9B···C13^v^	3.0500
C4···C7^iv^	3.528 (8)	H9B···C12	2.8500
C5···C9	3.574 (7)	H9B···H12	2.3300
C6···C1^iv^	3.509 (7)	H10A···H8A	2.4100
C7···O1	3.257 (10)	H10A···H16	2.3900
C7···C3^iv^	3.568 (9)	H10B···H25^ii^	2.4300
C7···C4^iv^	3.528 (8)	H10B···H8B	2.5500
C8···C2^iv^	3.597 (7)	H12···H9B	2.3300
C9···C5	3.574 (7)	H12···C25^vii^	3.0700
C17···O1	3.236 (13)	H12···C9	2.6900
C1···H18B	3.0900	H13···Br1^v^	3.1000
C2···H17B	2.9500	H14···HW1^v^	2.3300
C5···H9A	2.9900	H15···H21^v^	2.5400
C5···H8A	3.0600	H16···H10A	2.3900
C6···H9A	2.9700	H17A···O1	2.3800
C7···H18A	3.0700	H17A···H19A	2.5500
C8···H5	3.0200	H17A···H7	2.5700
C9···H12	2.6900	H17B···C2	2.9500
C11···H22^vi^	3.0200	H17B···H2	2.5600
C11···H24^ii^	2.9000	H17B···Br1^x^	3.1100
C12···H9A	2.9600	H18A···C21	2.9400
C12···H9B	2.8500	H18A···C7	3.0700
C13···H9B^v^	3.0500	H18B···C1	3.0900
C15···H24^ii^	3.0600	H19A···H17A	2.5500
C16···H24^ii^	2.8100	H19A···H21	2.5100
C17···H2	3.0200	H19B···H25	2.3800
C18···H23^vii^	3.0700	H21···H15^v^	2.5400
C20···H5^viii^	3.0900	H21···H19A	2.5100
C21···H18A	2.9400	H22···C11^viii^	3.0200
C25···H12^ix^	3.0700	H23···C18^ix^	3.0700
HW1···H14^v^	2.3300	H24···C11^x^	2.9000
HW1···Br1^iii^	3.04 (9)	H24···C16^x^	2.8100
H2···Br1^x^	3.1600	H24···C15^x^	3.0600
H2···H17B	2.5600	H25···H10B^x^	2.4300
H2···C17	3.0200	H25···H19B	2.3800
			
HW1—O1—HW2	105 (9)	N2—C8—H8A	109.00
C1—N1—C7	107.9 (3)	C9—C8—H8A	109.00
C1—N1—C17	126.5 (4)	C9—C8—H8B	109.00
C7—N1—C17	125.6 (4)	N2—C8—H8B	109.00
C6—N2—C7	108.1 (4)	H8A—C8—H8B	108.00
C6—N2—C8	126.3 (4)	C8—C9—H9B	109.00
C7—N2—C8	125.6 (4)	C10—C9—H9A	109.00
N1—C1—C6	106.7 (4)	C10—C9—H9B	109.00
C2—C1—C6	121.8 (4)	H9A—C9—H9B	108.00
N1—C1—C2	131.5 (4)	C8—C9—H9A	109.00
C1—C2—C3	115.9 (4)	C9—C10—H10A	108.00
C2—C3—C4	122.1 (5)	C9—C10—H10B	108.00
C3—C4—C5	122.2 (5)	C11—C10—H10B	108.00
C4—C5—C6	115.9 (4)	H10A—C10—H10B	107.00
N2—C6—C1	106.2 (4)	C11—C10—H10A	108.00
C1—C6—C5	122.1 (4)	C11—C12—H12	120.00
N2—C6—C5	131.8 (4)	C13—C12—H12	120.00
N1—C7—N2	111.2 (5)	C12—C13—H13	120.00
N2—C8—C9	111.1 (5)	C14—C13—H13	120.00
C8—C9—C10	112.5 (6)	C15—C14—H14	120.00
C9—C10—C11	117.0 (7)	C13—C14—H14	120.00
C10—C11—C12	123.8 (6)	C16—C15—H15	120.00
C12—C11—C16	117.7 (7)	C14—C15—H15	120.00
C10—C11—C16	118.5 (8)	C11—C16—H16	120.00
C11—C12—C13	120.6 (6)	C15—C16—H16	120.00
C12—C13—C14	120.2 (9)	N1—C17—H17B	110.00
C13—C14—C15	120.8 (9)	C18—C17—H17A	110.00
C14—C15—C16	120.2 (8)	N1—C17—H17A	110.00
C11—C16—C15	120.5 (9)	H17A—C17—H17B	108.00
N1—C17—C18	108.6 (4)	C18—C17—H17B	110.00
C17—C18—C19	113.8 (5)	C17—C18—H18A	109.00
C18—C19—C20	110.4 (6)	C17—C18—H18B	109.00
C19—C20—C21	120.7 (8)	C19—C18—H18B	109.00
C19—C20—C25	121.4 (6)	H18A—C18—H18B	108.00
C21—C20—C25	117.9 (8)	C19—C18—H18A	109.00
C20—C21—C22	120.0 (10)	C18—C19—H19B	110.00
C21—C22—C23	120.8 (9)	C20—C19—H19A	110.00
C22—C23—C24	118.9 (12)	C20—C19—H19B	110.00
C23—C24—C25	121.2 (12)	H19A—C19—H19B	108.00
C20—C25—C24	120.9 (8)	C18—C19—H19A	110.00
C3—C2—H2	122.00	C22—C21—H21	120.00
C1—C2—H2	122.00	C20—C21—H21	120.00
C2—C3—H3	119.00	C21—C22—H22	120.00
C4—C3—H3	119.00	C23—C22—H22	120.00
C5—C4—H4	119.00	C24—C23—H23	120.00
C3—C4—H4	119.00	C22—C23—H23	121.00
C4—C5—H5	122.00	C23—C24—H24	119.00
C6—C5—H5	122.00	C25—C24—H24	119.00
N2—C7—H7	124.00	C20—C25—H25	119.00
N1—C7—H7	124.00	C24—C25—H25	120.00
			
C7—N1—C1—C2	178.3 (6)	C4—C5—C6—C1	−0.6 (8)
C17—N1—C1—C2	−3.8 (8)	N2—C8—C9—C10	−177.5 (5)
C7—N1—C1—C6	0.2 (5)	C8—C9—C10—C11	173.4 (5)
C17—N1—C1—C6	178.1 (4)	C9—C10—C11—C12	24.9 (9)
C1—N1—C7—N2	0.3 (6)	C9—C10—C11—C16	−157.8 (7)
C17—N1—C7—N2	−177.7 (4)	C10—C11—C12—C13	174.1 (7)
C1—N1—C17—C18	−85.3 (5)	C12—C11—C16—C15	3.1 (13)
C7—N1—C17—C18	92.3 (7)	C16—C11—C12—C13	−3.2 (11)
C6—N2—C8—C9	72.9 (5)	C10—C11—C16—C15	−174.4 (9)
C7—N2—C8—C9	−107.4 (6)	C11—C12—C13—C14	1.0 (12)
C7—N2—C6—C5	−177.9 (6)	C12—C13—C14—C15	1.4 (14)
C8—N2—C6—C5	1.8 (9)	C13—C14—C15—C16	−1.5 (17)
C7—N2—C6—C1	0.6 (5)	C14—C15—C16—C11	−0.8 (16)
C8—N2—C6—C1	−179.7 (4)	N1—C17—C18—C19	−175.7 (5)
C6—N2—C7—N1	−0.6 (6)	C17—C18—C19—C20	−179.6 (5)
C8—N2—C7—N1	179.7 (4)	C18—C19—C20—C21	−81.0 (8)
N1—C1—C2—C3	−177.6 (5)	C18—C19—C20—C25	97.9 (7)
C2—C1—C6—C5	−0.1 (8)	C19—C20—C21—C22	173.4 (7)
N1—C1—C6—C5	178.3 (5)	C25—C20—C21—C22	−5.5 (11)
C6—C1—C2—C3	0.4 (8)	C19—C20—C25—C24	−177.4 (7)
C2—C1—C6—N2	−178.8 (5)	C21—C20—C25—C24	1.5 (10)
N1—C1—C6—N2	−0.5 (5)	C20—C21—C22—C23	4.6 (14)
C1—C2—C3—C4	0.1 (8)	C21—C22—C23—C24	0.6 (14)
C2—C3—C4—C5	−0.9 (9)	C22—C23—C24—C25	−4.8 (14)
C3—C4—C5—C6	1.1 (8)	C23—C24—C25—C20	3.8 (13)
C4—C5—C6—N2	177.8 (5)		

Symmetry codes: (i) −*x*, *y*−1/2, −*z*+3/2; (ii) *x*, −*y*+1/2, *z*−1/2; (iii) −*x*, *y*+1/2, −*z*+3/2; (iv) −*x*, −*y*, −*z*+2; (v) −*x*−1, −*y*, −*z*+1; (vi) *x*, *y*−1, *z*; (vii) −*x*−1, *y*−1/2, −*z*+3/2; (viii) *x*, *y*+1, *z*; (ix) −*x*−1, *y*+1/2, −*z*+3/2; (x) *x*, −*y*+1/2, *z*+1/2.

### Hydrogen-bond geometry (Å, °)

**Table:**

*D*—H···*A*	*D*—H	H···*A*	*D*···*A*	*D*—H···*A*
C7—H7···O1	0.93	2.50	3.257 (10)	139
C17—H17A···O1	0.97	2.38	3.236 (13)	148
C24—H24···Cg1^x^	0.93	2.84	3.771 (14)	176

Symmetry codes: (x) *x*, −*y*+1/2, *z*+1/2.
